# Supplementary material for: Acclimation of liverwort Marchantia polymorpha to physiological drought reveals important roles of antioxidant enzymes, proline and abscisic acid in land plant adaptation to osmotic stress
Source: PeerJ. 2021 Nov 10;9:e12419. doi: 10.7717/peerj.12419 (PMC8590393; doi:10.7717/peerj.12419)
Supplement: Supplemental Information 1 — The protein sequences were collected by BLAST search using the databases of Phytozome v13. The amino acid sequences were aligned by ClustalW program. [file peerj-09-12419-s001.pdf]

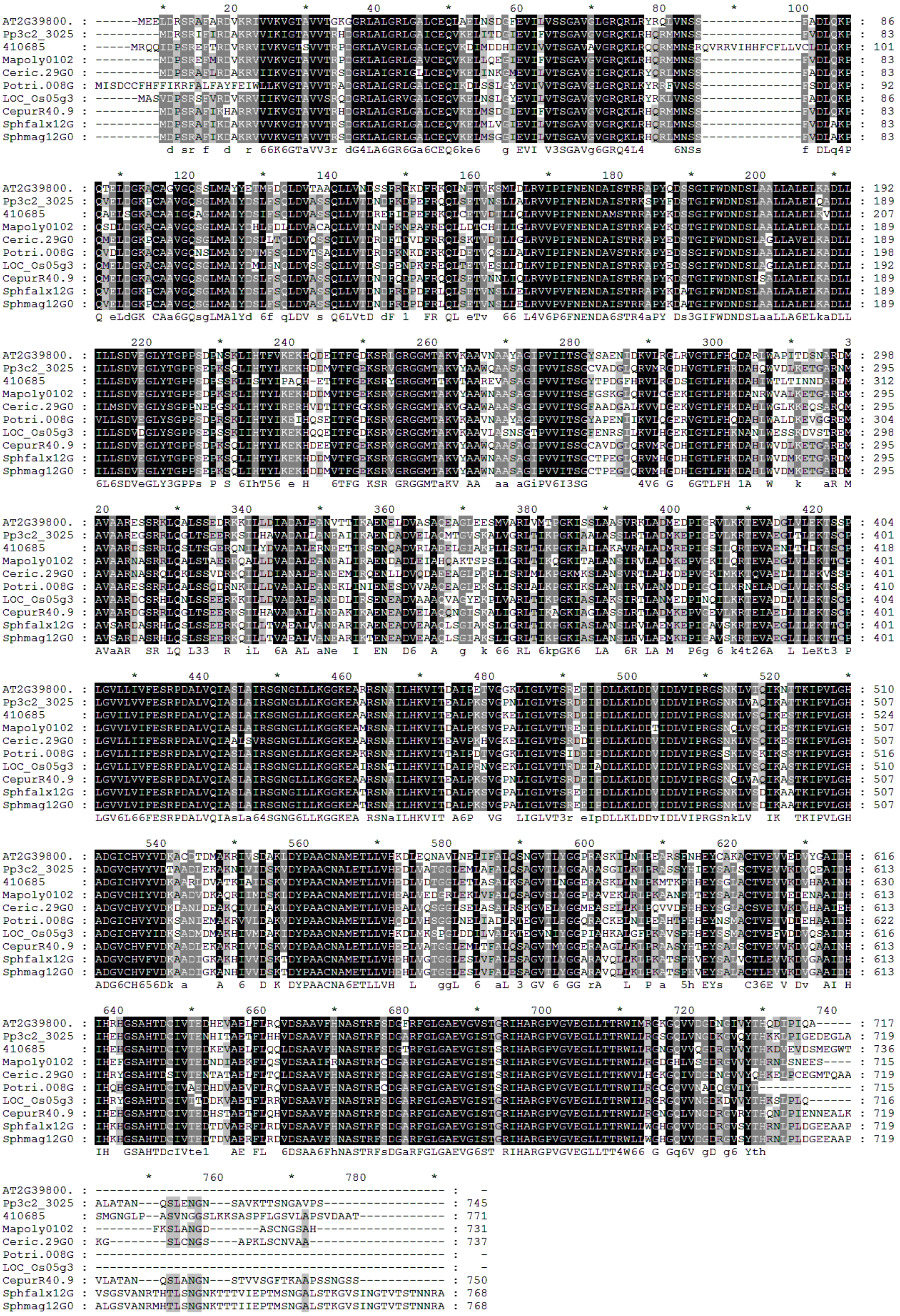

AT2G39800. : 86
Pp3c2_3025 : 83
410685 : 101
Mapoly0102 : 83
Ceric.29G0 : 83
Potri.008G : 92
LOC_Os05g3 : 86
CepurR40.9 : 83
Sphfalx12G : 83
Sphmag12G0 : 83
